# Barriers to Adolescents’ Adherence to Medical Advice after Metabolic Bariatric Surgery: A Statistical Analysis

**DOI:** 10.3390/jcm13061762

**Published:** 2024-03-19

**Authors:** Evia Shellac, Rachel Lev-Wiesel, Iris Shachar-Lavie, Arie Hadas, Adi Sela, Irit Halifa-Kurzman, Adi Bar-Eyal, Shlomit Shalitin, Dragan Kravarusic, Osher Cohen, Simona Tyroler, Orly Lavan, Silvana Fennig

**Affiliations:** 1 Department of Child and Adolescent Psychiatry, Schneider Children’s Medical Center of Israel, Petach-Tikva 4920235, Israelorlylavan84@gmail.com (O.L.); silvanaf@clalit.org.il (S.F.); 2 Faculty of Social Welfare and Health Sciences, School of Creative Arts Therapies, University of Haifa, Haifa 3498838, Israel; 3 Faculty of Medicine, Tel Aviv University, Tel Aviv 6997801, Israel; shlomits2@clalit.org.il (S.S.); dragank@clalit.org.il (D.K.); 4 Department of Surgery, Schneider Children’s Medical Center of Israel, Petach-Tikva 4920235, Israelsimonati@clalit.org.il (S.T.)

**Keywords:** metabolic bariatric surgery, adherence, adolescents, autonomy, family activities

## Abstract

**Background:** Adolescent obesity has markedly increased worldwide, and metabolic bariatric surgery is an effective treatment option. A major predictor of the outcomes of this procedure is adherence to post-surgery lifestyle changes and medical recommendations. While adolescents generally have more difficulty adhering to medical advice than adults, their failure to do so could adversely affect their physical and psychological health, the cost-effectiveness of medical care, and the results of clinical trials. To our knowledge, this is the first attempt to identify the characteristics associated with the adherence of adolescents and their families to medical advice after bariatric surgery. **Methods:** We investigated potential variables influencing adherence to medical advice in adolescents diagnosed with severe obesity enrolled in a nutritional and behavior-oriented bariatric program—a 3-month pre-surgical outpatient intervention and a 6-month post-surgical follow-up. The program monitored weight, program attendance, diet compliance, lifestyle changes, and daily activities. All participants and parents completed a standard battery of questionnaires, provided demographic information, and participated in a semi-structured interview about their lifestyle. **Results:** The study group consisted of 47 adolescents: 34 girls and 13 boys, aged 13–18 years. Over time, three groups emerged with different degrees of adherence—high, low, and delayed low adherence. The analyses showed that adolescents’ depression, autonomy, and independence from their family had strong, significant effects on adherence across the groups. **Conclusions:** Using adherence typologies, practitioners may be able to identify, predict, and tailor interventions to improve adolescent adherence to post-surgery recommendations. Parents have an important role in ensuring that adolescents undergoing metabolic bariatric surgery follow medical advice after the procedure.

## 1. Introduction

Pediatric obesity rates, particularly severe obesity among adolescents, continue to increase worldwide [1]. Some weight loss programs and lifestyle modifications have proven effective for sustained weight loss and preventing potential long-term conditions associated with chronic obesity. The effectiveness of drug treatment is unclear. Both metabolic bariatric surgery and drug treatment require adherence to radical lifestyle changes.

Metabolic bariatric surgery is a life-altering surgery for weight loss that reduces medical complications and can maintain weight loss. After bariatric surgery, patients must take supplements, have routine screenings, and commit to lifestyle changes [2]. Studies have shown that adolescent patients have poor rates of adherence to these measures [3], although the factors that promote such adherence are unknown [4].

Failure to follow these medical regimens can adversely affect patients’ physical and psychological health, the cost-effectiveness of medical care, clinical decisions, and the results of clinical trials. For adolescents with chronic health conditions, failure to follow these medical regimens is the primary cause of treatment failures and reduced quality-of-life [5,6].

Adolescents are less likely than adults to adhere to medical recommendations [7] due to psychological, physiological, sociological, and family-related factors. Data on metabolic bariatric surgery and adolescents’ adherence to such recommendations are limited. Therefore, this study seeks to fill the gap in the literature on this issue.

## 2. Materials and Methods

The study was conducted in an eating disorder ward at Children’s Medical Center in Israel. The institution’s IRB approved the study in accordance with the ethical standards in the 1964 Declaration of Helsinki and its later amendments.

The bariatric adolescent program treats the needs of severely obese adolescent candidates for metabolic bariatric surgery using a day treatment format. The multidisciplinary team includes a pediatric surgeon specializing in laparoscopic bariatric surgery, a pediatric endocrinologist, a medical management nurse, and a psychosocial and nutritional team. This team consists of a child and adolescent psychiatrist, a psychiatric nurse, a physical education teacher, a social worker, a dietician, psychotherapists, and social guides/mentors (undergraduate psychology students), with expertise in adolescent metabolic bariatric surgery.

### 2.1. The Psychosocial and Nutritional Program

The 9-month program is based on a daily treatment format: three months of pre-surgical weekly assessments and preparation, a multidisciplinary bariatric committee to approve the metabolic bariatric surgery itself, and six months of post-surgical day treatment follow-ups weekly or monthly. The day treatment program follows a highly structured format. Patients attend the unit once a week, from 8:00 a.m. to 2:00 p.m. They continue their routine life at home and at school while implementing the program’s recommendations. The weekly sessions in the 3-month pre-surgical phase include dietary and activity counseling, and family sessions. The parents participate in a weekly group session. There are also group therapy sessions and activities, including an art group therapy and a psychodrama group therapy, a physical activity group, and a nutrition group. The program educates the teenagers and their parents about metabolic bariatric surgery, the self-monitoring of food intake and daily activities, and lifestyle changes. It encourages the involvement of the entire family and lifestyle changes. The highly structured format is conceptualized as an open group treatment of approximately 10 pre- and post-metabolic bariatric surgery adolescents.

### 2.2. Participants

The study group consisted of 47 adolescents diagnosed with severe obesity and included 34 girls and 13 boys, aged 13–18 (mean 16.2, SD = 1.23), who were referred to the program by the hospital’s inpatient or outpatient pediatric units or a community pediatrician. Eligibility criteria, according to the Israeli Ministry of Health, were a body mass index (BMI; weight in kilograms/height in m^2^) > 35 with a serious physical complication amenable to improvement by weight reduction, or a BMI > 40 with less severe medical complications and previous failure to lose weight in intensive outpatient programs. Exclusion criteria were a medical, disease-related etiology for obesity, intellectual disability or inability to understand or cooperate with the program’s requirements, and a diagnosis of a severe non-stabilized psychiatric disorder at initial assessment that required acute intervention. Also excluded were patients whose caretakers refused to participate in the program.

### 2.3. Description of the Metabolic Bariatric Surgery Performed

All participants underwent a laparoscopic sleeve procedure followed by a 4-day inpatient hospitalization in the surgical ward. Laparoscopic sleeve gastrectomy (LSG) is an essential bariatric procedure performed in obese patients, which results in significant weight loss and has a positive impact on obesity-related diseases. The first laparoscopic procedure took place in 1999 and was conducted by Ren et al. [8]. Since then, sleeve gastrectomy has been gaining popularity as a method of treating obesity and as a subject of medical research [9]. LSG is a procedure in which the greater curvature is resected, reducing the volume of the stomach by about 80%, which leads to significant limitation of food intake. Additionally, removal of the stomach fundus reduces the number of cells producing ghrelin—the “hunger hormone”. The reduction in plasma ghrelin concentration promotes the feeling of satiety and restrains food intake [10].

### 2.4. Assessment Protocol

Patients were assessed at three time points: (T1) at admission, (T2) three months following enrollment in the program and immediately prior to surgery, and (T3) six months after the surgery. Patients completed a standard battery at all time points. Parents completed a standard battery only at T1 to obtain a more detailed and comprehensive assessment of their characteristics and mental health.

### 2.5. Measures

During the program, the team monitored the patients and parents weekly with regard to their compliance. The monitoring included scheduled visits with the adolescents, a parents’ support group, nutritional and vitamin recommendations, and the self-monitoring of food intake and physical activity. The patients’ battery included: (1) a demographic questionnaire; (2) the Beck Depression Inventory for assessing depression; (3) the Parental Bonding Instrument for assessing parenting and parental bonding; (4) a Social Adjustment Self-Report Scale in six areas in life; (5) FACES—the Family Adaptability and Cohesion Evaluation Questionnaire; (6) the Treatment Self-Regulation Questionnaire to measure autonomous and controlled reasons to engage and persist in a medical regiment concerning weight loss; (7) the Self-Efficacy and Parental Effectiveness in Eating Disorders Questionnaire, measuring coping with obesity; (8) the Morgan–Russell interview, with five scales assessing body weight, eating habits, menstruation, social adjustment, and professional and/or academic adjustment; (9) the self-report Social Adjustment Scale that measures social functioning over the past two weeks using four different scales; and (10) the Mood and Feelings Questionnaire (MFQ) to detect depressive symptoms.

### 2.6. Data Collection

Demographic information was obtained from the psychiatric assessment and semi-structured interview at T1. Information about medical and psychiatric co-severities and pharmacotherapies came from the psychiatric assessment at admission (reported by parents and adolescents) and medical records. Details about daily routines, social functioning, and patient and familial weight history were obtained primarily from the semi-structured interviews administered to patients and parents (T1). Daily food intake and activity details came from self-monitoring reports. Program attendance by adolescents and parents was noted at each visit.

### 2.7. Statistical Analysis

To identify latent profiles of adherence over time after metabolic bariatric surgery, we utilized a Group-Based Trajectory Modeling (GBTM) [9], using MPlus 8.4 software (Muthén and Muthén, 1998–2017, Los Angeles, CA, USA) [11]. This method enabled us to identify phenotype subgroups before surgery that might correspond to different patterns of changes in adherence after surgery. The variable used in this process was participants’ adherence (yes, no) in 11 time points after metabolic bariatric surgery. Growth Curve Modeling (GCM) was used to estimate whether a linear or quadratic model better fit the observed data based on weeks. The MLR estimator (maximum likelihood parameter estimates with standard errors and a chi-squared test statistic robust to non-normality and non-independence of observations) estimated GBTMs with 1–4 possible classes. To ensure true maximum likelihood estimation, we replicated the model using 100 random starts. The solutions were evaluated and compared based on fit statistics, interpretability, and theoretical considerations to determine the optimal number of latent classes [12]. A good model fit was indicated by a lower Bayesian information criterion (BIC), Akaike information criteria (AIC), a sample size-adjusted Bayesian information criterion (adjBIC), higher entropy, and a significant Lo–Mendell–Rubin likelihood ratio test (LMR-LRT) result and bootstrap likelihood ratio test (BLRT). The fit of the model indices with these indicators suggested that the profile solution had a good fit and was a credible classification [13]. Analyses were conducted in accordance with the guidelines of Frankfurt and colleagues [14] and reported according to the guidelines of Van De Schoot et al. [15]. Although the 4-group solution was superior to other models with respect to the adjBIC value, the LMR-LRT and BLRT tests were insignificant. In addition, one of the groups (moderate compliance) comprised only five people. Therefore, we selected the 3-group solution as the ideal one.

In the next step, we compared the three adherence groups in a series of measures appraised during children’s intake and before the bariatric surgery (approximately three months after the intake). These analyses were conducted to detect specific factors at intake and/or before surgery that are related to the pattern of adherence following the surgery. Specifically, the following measures were compared as reported by the parents at intake and the children at intake and before surgery: lifestyle (the family’s daily and weekend routines, family meals, family activities during the weekend, and the quality of the relationship with parents), mental status (depression, hypomania, obsession, and anxiety), social tendencies (relationship with the family, independence from the family, interpersonal relationships outside the family, social activities outside the family, and social functioning), social adjustment (employment, social life, extended family, major relationships, and parenting), functioning (physical, emotional, social, and academic), depressive symptoms, mood, family adaptability and cohesion, treatment motivation, self-efficacy, and parental effectiveness in dealing with eating disorders. A series of chi-squared tests for the independence of measures with Fisher’s exact test estimated the significance of the differences in the lifestyle measures; the rest of the measures were compared using Welch’s one-way analyses of variance with Tamhane post hoc analyses. Significance was adjusted using a False Discovery Rate (FDR) of 5% to account for multiple comparisons. We also conducted a between-within-subjects ANOVA to appraise changes in BMI as a function of adherence groups with time as the within-subject independence measure and adherence group as the between-subject independence measure. BMI was the only medical measure we appraised over time.

## 3. Results

### 3.1. Group-Based Trajectory Modeling

**Figure 1:**
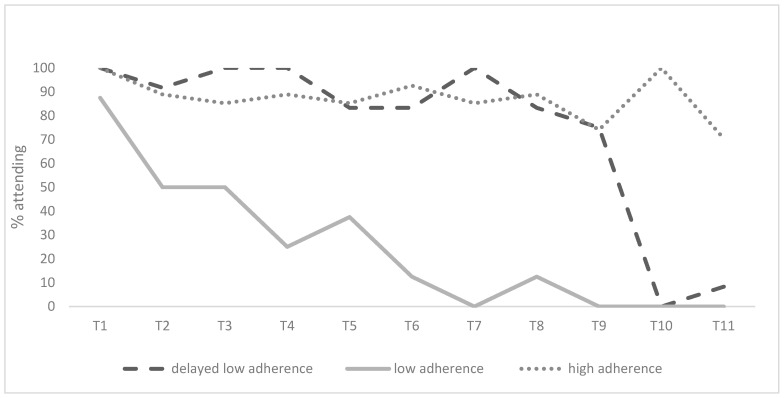
Trajectory of change in attending meetings after the metabolic bariatric surgery as a function of adherence groups.

**Figure 2:**
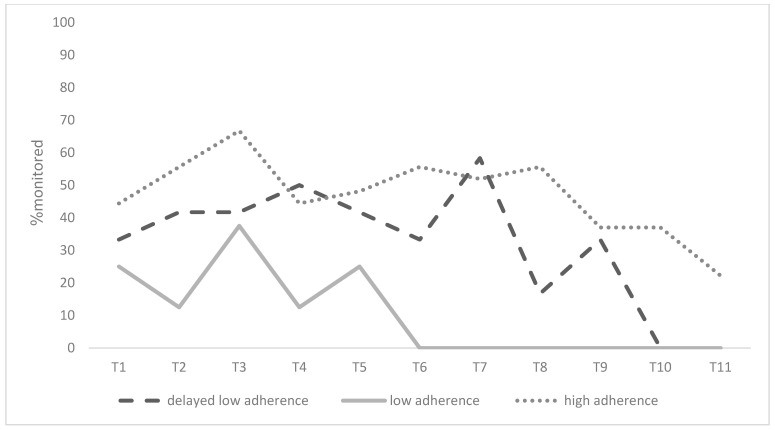
Trajectory of change in monitoring after the metabolic bariatric surgery as a function of adherence groups.

The GBTMs indicated that a three-group solution was ideal for detecting patterns of change in participants’ adherence over the course of 11 time points after the surgery. The three groups that were detected were “high adherence” (57.4%, *n* = 27), “low adherence” (17.0%, *n* = 8), and “delayed low adherence” (25.5%, *n* = 12), with the latter referring to high adherence post-surgery that dropped in subsequent weeks. Figure 1 and Figure 2 present the adherence trajectories of these groups with respect to attendance and monitoring across the 11 time points after the surgery, respectively.

### 3.2. Differences between Children’s Adherence Groups in Parental Reports at Intake

**Figure 3:**
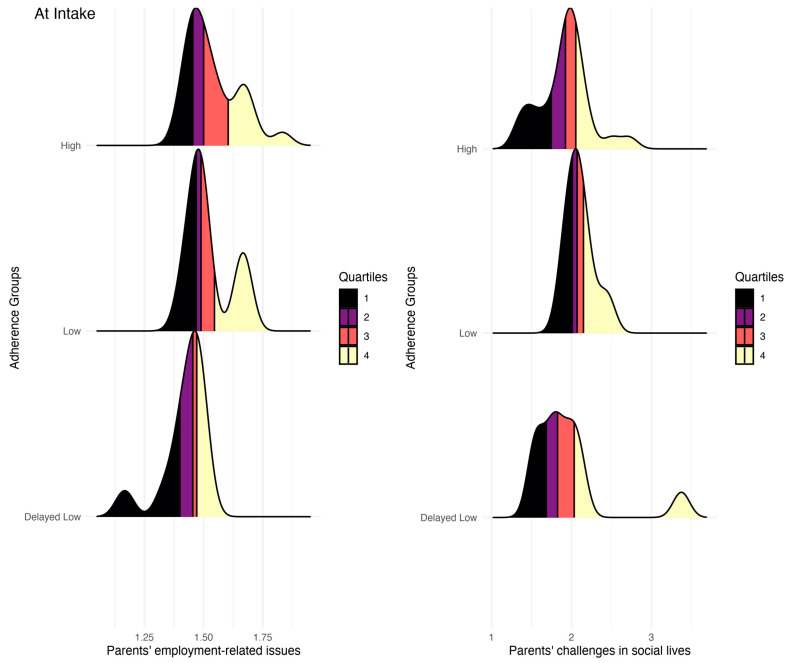
Differences in parents’ reports at intake on employment-related issues and challenges in their social lives.

The results are presented in Table 1 and Supplementary Table S1. The analysis identified two measures that remained significant after adjusting for false discovery rate (FDR). Specifically, it was found that parents in the “delayed low adherence” group experienced significantly more employment-related issues compared to parents of children with “high” adherence and faced more challenges in their social lives than parents of children with “low” adherence (all *p*-values < 0.05). Results are presented in Figure 3. Other findings were not statistically significant.

### 3.3. Differences between Children’s Adherence Groups in Children’s Reports at Intake

**Figure 4:**
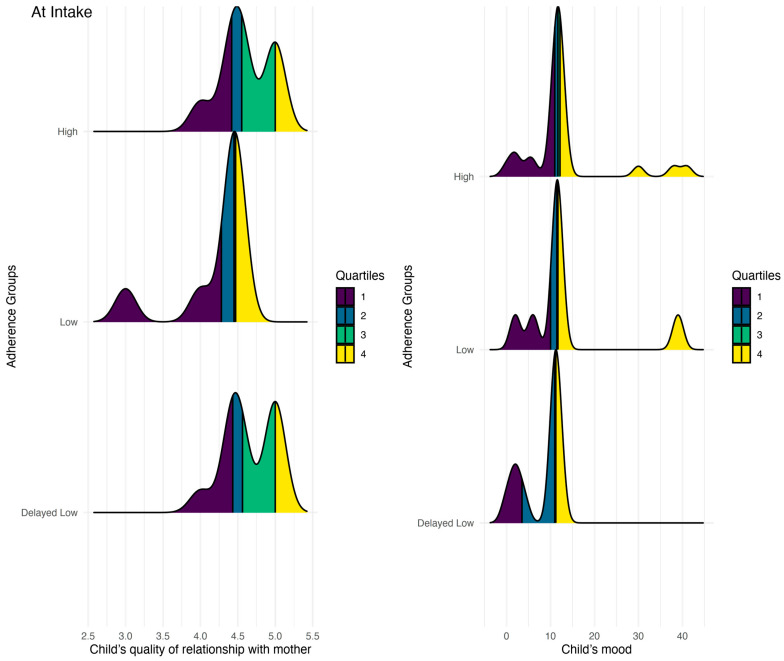
Differences in children’s reports at intake on quality of relationship with their mother and mood.

The results, presented in Table 2 and Supplementary Table S2a,b, demonstrate that at the time of intake, children with low adherence engaged in fewer family activities over the weekend and reported lower-quality relationships with their mothers compared to those with either high or delayed low adherence (all *p*-values < 0.05). Additionally, at intake, children with delayed low adherence exhibited a worse mood than those with high adherence (*p* < 0.05). Results are presented in Figure 4. Other findings were not statistically significant.

### 3.4. Differences between Children’s Adherence Groups in Children’s Reports before Surgery

**Figure 5:**
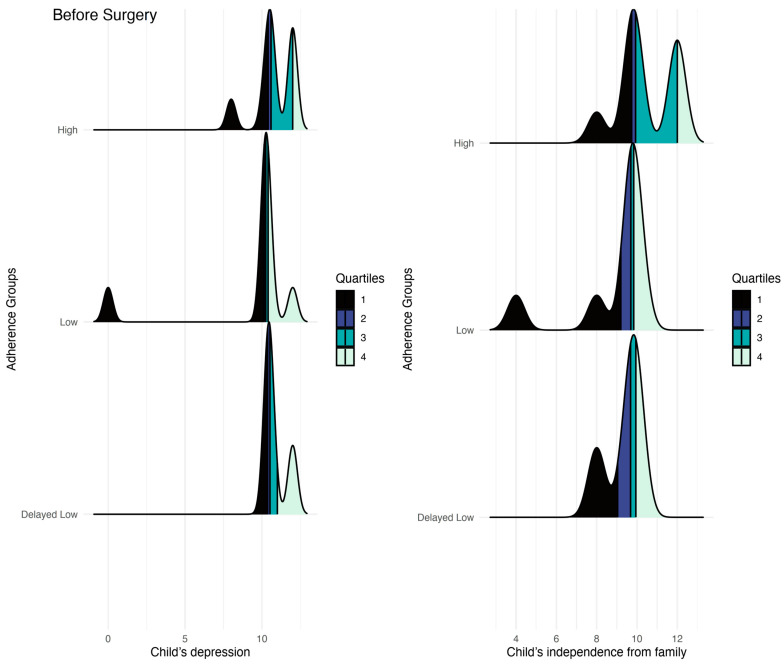
Differences in children’s reports before surgery on depression and independence from their families.

The results are detailed in Supplementary Table S2c,d. The analyses showed that, before undergoing bariatric surgery, children with low compliance experienced less depression compared to those with high or delayed low compliance (all *p*-values < 0.05). In contrast, children with high compliance demonstrated greater autonomy and independence from their families than those with low (marginally significant) and delayed low compliance (*p* < 0.05). Results are presented in Figure 5. Other findings were not statistically significant.

### 3.5. Differences in Children’s BMI

Aside from the differences in the psychological measures, we also examined whether the adherence groups differed in adolescents’ change in body mass index before and after the metabolic bariatric surgery. A between-within-subjects ANOVA revealed a significant main effect for time [*F*_(1,63)_ = 56.99, *p* = 2.25^−10^, *ε*^2^*_p_* = 0.47 (90% CI 0.32, 0.60)], indicating the participants’ BMI significantly decreased from an average of 46.8 (*SE* = 0.85) to 35.6 (*SE* = 1.22). This change in BMI was not dependent on the adherence group [i.e., no significant interaction; *F*_(1,63)_ = 0.03, *p* = 0.856, *ε*^2^*_p_* = 0.00 (90% CI 0.00, 0.00)].

## 4. Discussion

We examined the personal, interpersonal, and family characteristics that may predict differences in adolescents’ adherence to medical advice after metabolic bariatric surgery. We identified three major subgroups of phenotypes that might correspond to different patterns of change in adherence over time—high, low, and delayed low adherence. Findings indicate significant and strong effects of adolescents’ depression, autonomy, and family activities on differences in adherence to medical recommendations following the procedure.

Adolescents with low adherence had fewer depressive symptoms before the surgery than those with high and/or delayed low adherence. In contrast to the literature claiming that depression predicts poor adherence, we found that depression combined with a sense of autonomy and family activities actually increased adherence to the new lifestyle changes.

Most studies on the development of adolescent autonomy focus largely on theoretical and noncumulative aspects [16]. Although attempts have been made to differentiate among various aspects of autonomy (a state of being independent or self-governed, which frequently refers to behavioral, emotional, and cognitive domains), the concept of autonomy remains elusive [17]. Autonomy is considered a desirable state of being, in which the individual covets independence and the freedom to make choices. The challenge for adolescents is to maintain their connection with their family and society while becoming autonomous [18].

Research on issues of autonomy and independent decision-making in socioemotional health and peer and parental relationships found that autonomy has a role in how adolescents make choices and engage in health-related behaviors. For autonomy to develop, adolescents should be ready to assume more responsibility for health-related behaviors and parents should be ready to relinquish responsibility for managing these behaviors when they believe their child is capable [19]. If the development of autonomy, responsibility, and sound decision-making impact adolescents’ health, practitioners must approach them as a complex and whole entity—considering their social, emotional, developmental status, familial functioning, environmental contexts, and lifestyles. Thus, autonomy cannot be isolated from how adolescents practice either negative or positive compliance.

The link between autonomy and adherence is stressed in the self-determination theory. This theory is concerned with the motivations that affect people’s choices, regardless of external influences and interference [20]. It is the only motivation theory applied to the domains of health, education, work, and sports that identifies autonomy as a human need. Once this need is supported, autonomous forms of behavioral regulation occur, which are particularly critical for individuals learning to self-manage their obesity by improving their adherence to medical recommendations [21].

Although the relationship between autonomy and adherence has not been clearly established, we found that adolescents who followed medical advice had a significantly stronger sense of autonomy than those with low or delayed low compliance. Austin et al. (2011) and Bruzzese et al. (2014) emphasized the relationship between autonomy and adherence to treatment in their studies on adolescents with chronic illnesses [22,23]. It is also important to understand this relationship in the context of adolescents’ metabolic bariatric surgery due to differences in the complexity of care.

The tendency in studies on adolescents’ adherence to medical recommendations following metabolic bariatric surgery is to focus on the patient, despite our understanding that the natural supportive environment of adolescents is their parents. Our results accord with previous studies reporting that relatives have an effect on patients’ undergoing metabolic bariatric surgery and their adherence to subsequent medical recommendations [24]. Previous literature confirmed a correlation between having family members who promoted physical activity, and the physical activity of patients and family members [25]. Nevertheless, despite the potential role of family activities in promoting adherence to a healthier lifestyle and optimizing outcomes following surgery, to our knowledge, no study has investigated its relationship in the metabolic bariatric surgery field.

Parents may have an enormous influence on their adolescents’ behaviors. In order to increase compliance, clinical staff should work with parents and adolescents to raise the level of parental support. Thus, understanding the scope of this influence may help increase adolescents’ adherence to medical recommendations after bariatric surgery.

According to our research, family activities and developing a sense of autonomy may increase the likelihood that adolescents will adhere to the lifestyle changes required after bariatric surgery. These findings highlight the influence of parents and raise the question of moving the focus of interventions from the adolescent to the parents. Accordingly, involving parents in the post-operative appointments, assessing their concerns and struggles with the new lifestyle of the adolescent after the bariatric surgery, and encouraging positive attitudes might promote compliant behaviors. The parents’ ability to provide the adolescents with autonomy and independence is a vital resource. Therefore, healthcare providers need to be supportive of the parents and family if they are to maximize patient compliance.

We still have not identified the mechanisms through which family support leads to better adherence, and additional investigation in this regard is required. In this framework, we did not study the relationship between specific parent behaviors and other factors, such as parenting style. Exploring this interaction might provide more insights into the relationship between autonomy and independence from the family with regard to adolescents’ adherence to medical recommendations after bariatric surgery.

On a theoretical level, the significance of the current research is our understanding of the relationship between adolescent patients, their family, and their adherence to medical advice. On a practical level, our results provide practitioners with guidelines for appropriate patient education and suitable psychological pre -and post-operative methods for increasing the likelihood of compliance.

Despite the complexities of modern healthcare, including advanced technologies and capacities for direct intervention, human behavior still plays a critical role in health outcomes and shapes the efficacy of most treatments. Therefore, healthcare professionals can improve their efficacy by supporting parents in addition to supporting the adolescents’ psychological needs for autonomy. By doing so, they are not only enhancing the importance of patient compliance, but also emphasizing the ethical ideals of promoting patient autonomy and responsibility in healthcare decision-making and interventions.

## 5. Limitations

One of the limitations of our study is the use of self-reported answers. The respondents might have exaggerated their answers, been too embarrassed to reveal private details, or suffered from social desirability bias. In addition, the close monitoring of patients and caregivers may also serve as a source of bias (the Hawthorne effect). Other limitations are the small sample size and the length of time following the surgery when we measured the participants’ adherence.

However, despite these limitations, this study contributes to the knowledge about adolescent patients’ adherence to medical advice following metabolic bariatric surgery and is an important first step in further research. Lastly, this study began and ended before 2019 and before the outbreak of COVID-19. To the best of our knowledge, there are no studies on the current situation and its impact on adherence to medical advice following metabolic bariatric surgery. Future studies should focus on the pandemic’s impact on adolescent patients’ adherence to medical recommendations following such procedures.

## Figures and Tables

**Table 1:** Percentage of lifestyle indices by adherence group.

		Compliance				
		Low	Delayed Low	High	*χ*²	*Φ*
Family daily routine	No	46.25	40.83	48.89	3.32	0.18
	Yes	28.75	25	32.59		
	Partially	25	34.17	18.52		
Family weekend routine	No	20	24.17	32.96	4.23	0.21
	Yes	36.25	57.14	44.81		
	Partially	43.75	21.67	21.85		
Family meals	No	25	11.67	25.19	2.12	0.2
	Yes	75	88.33	74.81		
Family activities on weekends	No	28.75	30	33.33	1.49	0.16
	Yes	71.25	70	66.67		

Note. *φ* = effect size.

**Table 2:** Percentage of lifestyle indices by adherence group.

		Compliance				
		Low	Delayed Low	High	*χ*²	*Φ*
Family daily routine	No	12.5	41.7	37	3.1	0.18
	Yes	75	58.3	55.6		
	Partially	12.5	0	7.4		
Family weekend routine	No	12.5	8.3	25.9	2.96	0.2
	Yes	87.5	75	66.7		
	Partially	0	16.7	7.4		
Family meals	No	0	0	7.4	0.97	0.18
	Yes	100	100	92.6		
Family activities on weekends	No	62.5	16.7	25.9	4.73 *	0.34
	Yes	37.5	83.3	74.1		

Note. *φ* = effect size. * *p* < 0.05.

## Data Availability

Data is unavailable due to privacy restrictions.

## Supplementary Materials

The following supporting information can be downloaded at: https://www.mdpi.com/article/10.3390/jcm13061762/s1, Table S1: Parental report; Table S2: Children’s report.
