# Supplementary material for: Barriers to Adolescents’ Adherence to Medical Advice after Metabolic Bariatric Surgery: A Statistical Analysis
Source: J Clin Med. 2024 Mar 19;13(6):1762. doi: 10.3390/jcm13061762 (PMC10970738; doi:10.3390/jcm13061762)
Supplement: Supplementary file 1 [file jcm-13-01762-s001.zip › jcm-2644633-supplementary.pdf]

Journal of
Clinical Medicine

MDPI

Supplementary Table S1 – Parental report

Intake

Table S1a. Percentage of lifestyle indices at intake as a function of adherence groups.

		Adherence				
		Low	Delayed low	High	χ^2	φ
Family daily routine	No	46.25	40.83	48.89	3.32	0.18
	Yes	28.75	25.00	32.59		
	Partially	25.00	34.17	18.52		
Family weekend routine	No	20.00	24.17	32.96	4.23	0.21
	Yes	36.25	57.14	44.81		
	Partially	43.75	21.67	21.85		
Family meals	No	25.00	11.67	25.19	2.12	0.20
	Yes	75.00	88.33	74.81		
Family activities weekend	No	28.75	30.00	33.33	1.49	0.16
	Yes	71.25	70.00	66.67		

Note. φ = effect size. .

Table S1b. Means, standard deviations, statistics, and effect sizes for the differences in parents' perception of their children's condition and their own condition.

	Low		Delayed low		High			
	M	*SD*	*M*	*SD*	*M*	*SD*	$F_{(2,44)}$	η^2
Child's quality of relationship with mother	4.40	0.59	4.61	0.39	4.60	0.33	0.43	0.04
Child's quality of relationship with father	3.31	0.82	3.44	1.34	3.35	1.09	0.04	0.00
Child's depression	10.18	1.13	10.21	1.66	9.09	2.50	1.80	0.06
Child's hypomania	11.08	0.58	11.46	0.67	10.64	2.10	2.00	0.05
Child's obsession	10.69	0.82	10.58	1.48	10.06	2.40	0.67	0.02
Child's anxiety	10.90	0.68	10.78	1.45	10.73	1.72	0.09	0.00
Child's relationship with family	10.03	2.49	11.47	0.66	10.81	1.73	2.40	0.07
Child's independence from family	9.05	2.25	8.91	2.70	9.89	2.69	0.70	0.03
Child's interpersonal relationships	8.69	2.20	9.15	2.81	9.17	2.33	0.15	0.01
Child's social activities	5.82	2.54	5.49	4.14	4.67	3.61	0.55	0.02
Child's social functioning (e5)	10.08	1.08	9.58	3.34	10.59	1.30	0.96	0.05
Child's physical functioning	1.86	0.35	1.72	0.70	1.87	0.71	0.21	0.01
Child's emotional functioning	1.15	0.36	1.24	0.46	1.50	0.63	2.01	0.07
Child's social functioning (SAS)	1.08	0.56	1.45	0.74	1.33	0.64	0.87	0.03
Child's academic functioning	1.34	0.31	1.48	0.68	1.42	0.61	0.25	0.01
Child's depressive symptoms	3.57	0.22	2.73	1.22	3.64	2.11	2.63	0.05
Child's motivation for treatment	3.43	1.11	3.58	1.30	3.71	0.91	0.21	0.01
Parents' employment adjustment	1.52	0.10	1.42	0.09	1.54	0.16	5.72*	0.20
Parents' social life adjustment	2.10	0.17	1.81	0.49	1.91	0.31	5.40*	0.11
Parents' extended family adjustment	1.48	0.13	1.56	0.27	1.59	0.32	1.16	0.02

Parents' major relationships adjustment	1.97	0.09	1.98	0.35	1.96	0.81	0.00	0.00
Parents' parenting adjustment	1.54	0.20	1.45	0.37	1.44	0.30	0.61	0.02
Parents' familial adjustment	1.91	0.24	2.13	0.42	1.92	0.35	1.31	0.07
Parents' mood	3.27	1.34	3.63	1.25	4.17	3.45	0.60	0.02
Parents' family adaptability and cohesion	2.57	0.51	2.85	0.16	2.74	0.37	1.60	0.06
Parents' self-efficacy and parental effectiveness	7.92	0.54	7.93	0.55	7.67	0.67	0.98	0.04

Note. * $p < .05$.

Supplementary Table S2 – Children's report

Intake

Table 2a. Percentage of lifestyle indices at intake as a function of adherence groups.

		Adherence				
		Low	Delayed low	High	χ^2	φ
Family daily routine	No	12.5	41.7	37.0	3.10	0.18
	Yes	75.0	58.3	55.6		
	Partially	12.5	0.0	7.4		
Family weekend routine	No	12.5	8.3	25.9	2.96	0.20
	Yes	87.5	75.0	66.7		
	Partially	0.0	16.7	7.4		
Family meals	No	0.0	0.0	7.4	0.97	0.18
	Yes	100.0	100.0	92.6		
Family activities weekend	No	62.5	16.7	25.9	4.73*	0.34
	Yes	37.5	83.3	74.1		

Note. φ = effect size. * $p < .05$.

Table 2b. Means, standard deviations, statistics, and effect sizes for the differences in children's condition in the intake for bariatric surgery.

	Low		Delayed low		High			
	M	*SD*	*M*	*SD*	*M*	*SD*	$F_{(2,44)}$	η^2
Child's quality of relationship with mother	4.21	0.52	4.65	0.34	4.60	0.33	4.07*	0.16
Child's quality of relationship with father	3.65	0.31	3.92	1.07	3.71	1.01	0.33	0.01
Child's depression	8.30	1.74	9.58	2.30	8.92	1.98	0.97	0.04
Child's hypomania	11.43	0.36	11.60	0.43	11.14	1.20	1.52	0.04
Child's obsession	10.63	0.86	10.76	1.25	9.80	2.38	1.49	0.05
Child's anxiety	9.81	1.09	10.58	1.39	9.56	2.35	1.60	0.05
Child's relationship with family	10.46	1.13	11.26	0.78	10.59	2.10	1.99	0.03
Child's independence from family	9.90	1.30	9.22	2.21	9.07	2.53	0.84	0.02
Child's interpersonal relationships	8.95	2.23	9.67	2.29	9.24	3.02	0.25	0.01
Child's social activities	5.39	2.31	6.95	4.32	5.57	3.42	0.57	0.03
Child's social functioning (e5)	9.05	2.24	9.12	3.62	9.86	1.42	0.63	0.03

Child's physical functioning	1.89	0.44	1.50	0.76	1.81	0.57	1.06	0.06
Child's emotional functioning	1.21	0.59	0.94	0.70	1.20	0.75	0.61	0.03
Child's social functioning (SAS)	1.09	0.88	1.16	0.70	1.05	0.57	0.11	0.01
Child's academic functioning	1.38	0.77	1.49	1.00	1.50	0.63	0.08	0.00
Child's depressive symptoms	13.42	7.14	9.78	5.38	11.11	4.40	0.74	0.05
Child's attachment to mother	2.16	0.89	2.44	0.19	2.39	0.53	0.43	0.03
Child's attachment to mother	2.41	0.16	2.52	0.28	2.42	0.20	0.71	0.04
Child's mood	13.09	11.06	8.17	4.65	14.25	9.33	3.71*	0.09
Child's harassment	7.30	2.60	11.67	8.70	8.05	8.73	1.31	0.04
Child's cyberbullying	1.45	1.56	1.14	1.13	1.36	2.01	0.15	0.00
Child's bullying	10.79	0.27	11.43	1.06	10.60	1.70	2.18	0.06
Child's help seeking because of bullying	36.81	4.59	34.48	5.42	34.37	8.27	0.71	0.02

Note. * $p < .05$.

Before Surgery

Table 2c. Percentage of lifestyle indices at intake as a function of adherence groups.

		Adherence				
		Low	Delayed low	High	χ^2	φ
Family daily routine	No	12.5	25.0	29.8	3.18	0.21
	Yes	87.5	75.0	66.0		
	Partially	0.0	0.0	4.3		
Family weekend routine	No	0.0	16.7	29.6	4.42	0.22
	Yes	100.0	83.3	66.7		
	Partially	0.0	0.0	3.7		
Family meals	No	0.0	0.0	3.7	1.17	0.13
	Yes	100.0	100.0	96.3		
Family activities weekend	No	37.5	16.7	18.5	1.60	0.18
	Yes	62.5	83.3	81.5		

Note. φ = effect size. .

Table 2d. Means, standard deviations, statistics, and effect sizes for the differences in children's condition before bariatric surgery.

	Low		Delayed low		High			
	M	*SD*	*M*	*SD*	*M*	*SD*	$F_{(2,44)}$	η^2
Child's quality of relationship with mother	4.75	0.16	4.76	0.15	4.71	0.30	0.20	0.01
Child's quality of relationship with father	3.84	0.73	3.37	0.88	3.45	0.91	0.95	0.03
Child's depression	8.81	3.89	10.84	0.71	10.77	1.24	3.69*	0.15
Child's hypomania	9.18	3.76	10.68	0.83	10.50	1.74	0.64	0.06
Child's obsession	9.57	2.35	10.45	0.96	9.91	1.42	1.15	0.04
Child's anxiety	9.21	2.27	9.62	1.19	9.49	2.09	0.12	0.00
Child's relationship with family	11.59	0.27	11.64	0.23	11.61	0.76	0.10	0.00
Child's independence from family	8.87	2.08	9.37	0.85	10.43	1.36	5.09*	0.19

Child's interpersonal relationships	10.56	0.91	10.52	0.91	9.76	2.85	0.85	0.03
Child's social activities	8.52	1.45	8.26	1.23	7.71	3.68	0.41	0.01
Child's social functioning (e5)	10.37	1.12	10.83	0.74	10.55	2.30	0.57	0.01
Child's physical functioning	1.42	0.33	1.19	0.33	1.31	0.52	1.18	0.03
Child's emotional functioning	0.76	0.76	0.63	0.39	0.85	0.64	0.82	0.02
Child's social functioning (SAS)	0.55	0.47	0.69	0.30	0.83	0.61	1.00	0.04
Child's academic functioning	0.98	0.55	1.06	0.51	1.17	0.83	0.29	0.01
Child's depressive symptoms	6.07	0.44	5.06	1.98	6.36	3.87	1.51	0.03
Child's mood	6.51	7.78	4.05	2.31	5.86	4.61	1.48	0.03
Child's harassment	6.62	2.24	6.44	2.15	8.46	8.01	0.73	0.02
Child's cyberbullying	3.07	1.27	2.43	1.49	3.89	4.84	1.20	0.03
Child's bullying	21.22	12.89	27.11	5.18	25.11	8.45	0.93	0.05
Child's help seeking because of bullying	8.91	0.38	9.09	0.13	9.13	0.79	0.83	0.02

Note. * $p < .05$.
